# Molecular and Physio-Biochemical Characterization of Cotton Species for Assessing Drought Stress Tolerance

**DOI:** 10.3390/ijms19092636

**Published:** 2018-09-06

**Authors:** Md Mosfeq-Ul Hasan, Fanglu Ma, Zakaria Hossain Prodhan, Feng Li, Hao Shen, Yadong Chen, Xuede Wang

**Affiliations:** 1 Institute of Crop Science, College of Agriculture and Biotechnology, Zijingang Campus, Zhejiang University, Hangzhou 310058, China; hasanmd12@hotmail.com (M.M.-U.H.); 21516038@zju.edu.cn (F.M.); rajugenetics2003@gmail.com (Z.H.P.); seawind-1@zju.edu.cn (F.L.); shenhao1721@163.com (H.S.); sdaucyd@163.com (Y.C.); 2 Examination Control Section, Hajee Mohammad Danesh Science and Technology University, Dinajpur 5200, Bangladesh

**Keywords:** drought, drought tolerance, abiotic stress, antioxidant defense, oxidative stress, cotton

## Abstract

Drought stress significantly limits cotton growth and production due to the necessity of water at every stage of crop growth. Hence, it is essential to identify tolerant genetic resources and understand the mechanisms of drought tolerance in economically and socially important plants such as cotton. In this study, molecular and physio-biochemical investigations were conducted by analyzing different parameters by following standard protocols in three different cotton species, namely TM-1 (*Gossypium hirsutum*), Zhongmian-16 (*Gossypium arboreum*), and Pima4-S (*Gossypium barbadense*). Drought stress significantly decreased plant growth, chlorophyll content, net photosynthetic rate (*P*_n_), stomatal conductance (*Gs*), maximum photochemical efficiency of PSII (*Fv*/*Fm*), and relative water content. TM-1 resulted in more tolerance than the other two species. The accumulation of proline, soluble proteins, soluble sugars, hydrogen peroxide (H_2_O_2_), and superoxide radicals (O_2_^•^^−^) increased significantly in TM-1. In addition, TM-1 maintained the integrity of the chloroplast structure under drought conditions. The relative expression level of drought-responsive genes including coding for transcription factors and other regulatory proteins or enzymes controlling genes (*ERF*, *ERFB*, *DREB*, *WRKY6*, *ZFP1*, *FeSOD*, *CuZnSOD*, *MAPKKK17*, *P5CR*, and *PRP5*) were higher in TM-1 under drought, conferring a more tolerant status than in Zhongmian-16 and Pima4-S. The findings of this research could be utilized for predicting a tolerant cotton genotype as well as evaluating prospective cotton species in the variety development program.

## 1. Introduction

Drought is one of the most critical abiotic stresses that limit crop growth and productivity worldwide. Drought is considered a multidimensional stress that leads to changes in the physiological, morphological, ecological, biochemical, and molecular characteristics of plants. The symptoms of drought stress also vary with the plant species, developmental stages, growth conditions, and environmental factors [1,2]. Drought stress inhibits plant growth and development [3] but enables root length proliferation to acquire water from the deep soil and tolerate the stress [4,5]. The root/shoot ratio also increases, indicating water acclimatization and enhanced tolerance [6,7]. Decreased shoot length is observed due to the blockage of vascular tissue vessels and a reduction in cell elongation [8]. Generally, drought symptoms are mostly observed in the leaves of plants showing loss of turgor, drooping, wilting, etiolation, yellowing, and premature downfall [9,10,11]. The photosynthetic rate was found to decrease under drought conditions in different plant species [12]. Plants grown under drought conditions have lower stomatal conductance, reduced CO_2_ fixation, and decreased photosynthesis, which result in reduced growth and yield of plants. Severe drought stress also inhibits the photosynthesis of plants by causing changes in the chlorophyll content and damaging the photosynthetic apparatus [13]. Drought stress caused a large decline in chlorophyll a, chlorophyll b, and total chlorophyll content in sunflower varieties [14]. Drought also induces osmotic stress, oxidative damage, stomatal closure, damage to cellular structures, as well as a decrease in gas exchange rates [15,16].

Generally, drought stress induces the accumulation of reactive oxygen species (ROS) in plants and breaks cellular physiological homeostasis [17,18,19]. ROS, including superoxide anion radical (O_2_^•−^), hydrogen peroxide (H_2_O_2_), singlet oxygen (^1^O_2_), hydroxyl radical (HO^•^), nitric oxide (NO^•^), peroxyl radical (ROO^•^), ozone (O_3_), and hypochlorous acid (HOCl), interact with a number of other molecules and metabolites, such as photosynthetic pigments (chlorophyll a, chlorophyll b, carotenoids, etc.), proteins, lipids, and other crucial cellular particles, leading to an increase of damaging processes [20,21]. ROS also cause oxidative impairment at the cellular level [22]. However, antioxidant enzymes, including some isoenzymes in different cell compartments (cytosol, chloroplasts, and mitochondria), control cellular ROS levels during stress conditions [23]. Drought stress causes a significant increase (5% probability level) in Na^+^ and K^+^ concentrations [24] and a decrease in Zn and Cu concentrations [25]. In addition, increasing concentrations of Ca, Mn, and Fe were observed in the shoots [25].

The accumulation of proline in the tissues of numerous plant species is regarded as a common response to drought as well as other types of stresses [26,27]. Proline is produced from glutamic acid using the enzyme Δ^1^-pyrroline-5-carboxylate synthetase (P5CS) and pyrroline-5-carboxylate reductase [28,29]. Proline is a compatible osmolyte that controls osmotic regulation and alleviates stress in cell membranes. It also acts as a protective agent for enzymes’ function and as a free radical scavenger [30,31]. A higher proline accumulation was reported in drought-tolerant species of cotton, tall fescue, and wheat [32,33]. Hanson et al. [34] mentioned that the accumulation of proline is a symptom of leaf dryness, indicative of stress susceptibility. However, other researchers have demonstrated a positive link between the levels of proline accumulation in leaves and the resistance to stress in drought-treated barley species [25].

Drought also induced expression of drought-responsive genes including coding for transcription factors and other regulatory proteins or enzymes controlling genes such as ethylene responsive factor (*ERF*), ethylene responsive factor binding (*EREB*) [35], dehydration responsive element binding protein (*DREB*) [36], WRKY transcription factor 6 (*WRKY6*) [37], putative CCCH-type zinc finger transcription factor (*ZFP1*) [38], FeSOD and copper/zinc superoxide dismutase (*SOD1*) [39], mitogen-activated protein kinase kinase kinase 17 (*MAPKKK17*) [40], Δ^1^-pyrroline-5-carboxylase reductase (*Δ^1^P5CR*) [41], and proline-rich protein (*PRP5*) genes in different plant species. These genes could be utilized for characterizing drought stress-tolerant cotton genotypes.

Cotton is one of the most valuable crops for providing natural fibers for the textile industry globally. China, the United States, India, Pakistan, Uzbekistan, Turkey, and Brazil are the seven largest producers of cotton worldwide, while China, the United States, the Franc Zone of Africa, Uzbekistan, Australia, and India are the five leading exporters [42]. China, the United States, and India provide most of the world’s cotton; more than 15.95 million metric tons of cotton as lint and 29.26 million metric tons of cotton as seeds were exported in 2014 (FAOSTAT, www.faostat.fao.org). The productivity of cotton is detrimentally affected by biotic and abiotic stresses, such as fungi, harmful insects, drought, and soil salinity. Among abiotic stresses, drought was found to be the most serious stress that reduced cotton yields significantly at the experimental fields of the Agriculture Research Institute, Tandojam, Pakistan during the year 2007 [42,43].

Researchers are trying to identify drought-tolerant cotton germplasm or genotype based on morphological, biochemical, and physiological parameters to compensate for yield losses and improve productivity; however, studies are limited to the *Gossypium hirsutum* species, occasionally focusing on the *Gossypium herbaceum* or *Gossypium arboreum* species [44,45]. Some research tried to identify candidate genes related to drought stress in cotton [46,47] and, recently, an extensive review on the mechanism of drought tolerance in cotton [48] has been published. However, a complete elucidation of the morphological, physiological, biochemical, and molecular performance by which different cotton species respond to drought is still lacking and represents a challenge for plant biologists.

In the present study, the performance of the three cotton species *G. hirsutum* (TM-1), *G. arborium* (Zhongmian-16), and *G. barbadense* (Pima4-S) in terms of their morpho-physiological traits (plant height, root length, total fresh weight, total dry weight, relative water content, gas exchange, photosynthesis, stomatal conductance, intercellular CO_2_, and water use efficiency, WUE), biochemical properties (proline, soluble proteins, and soluble sugars content), and antioxidant activity (SOD, POD, APX, CAT, MDA, H_2_O_2_, and O_2_) were examined under drought conditions. The relative expression level of drought-responsive genes, coding for transcription factors and other regulatory proteins or enzymes controlling genes such as ethylene responsive factor (*ERF*), dehydration responsive element-binding protein (*DREB*), WRKY transcription factor 6 (*WRKY6*), putative CCCH-type zinc finger transcription factor (*ZFP1*), FeSOD, copper/zinc superoxide dismutase (*SOD1*), mitogen-activated protein kinase kinase kinase 17 (*MAPKKK17*), Δ^1^-pyrroline-5-carboxylase reductase (*Δ^1^P5CR*) and proline-rich protein (*PRP5*) genes was also analyzed for characterizing drought stress tolerant cotton species. The species *G. hirsutum* was found to be more tolerant than the other two species.

## 2. Results and Discussion

### 2.1. The Effect of Drought on Growth Parameters and Biomass Accumulation

**Figure 1:**
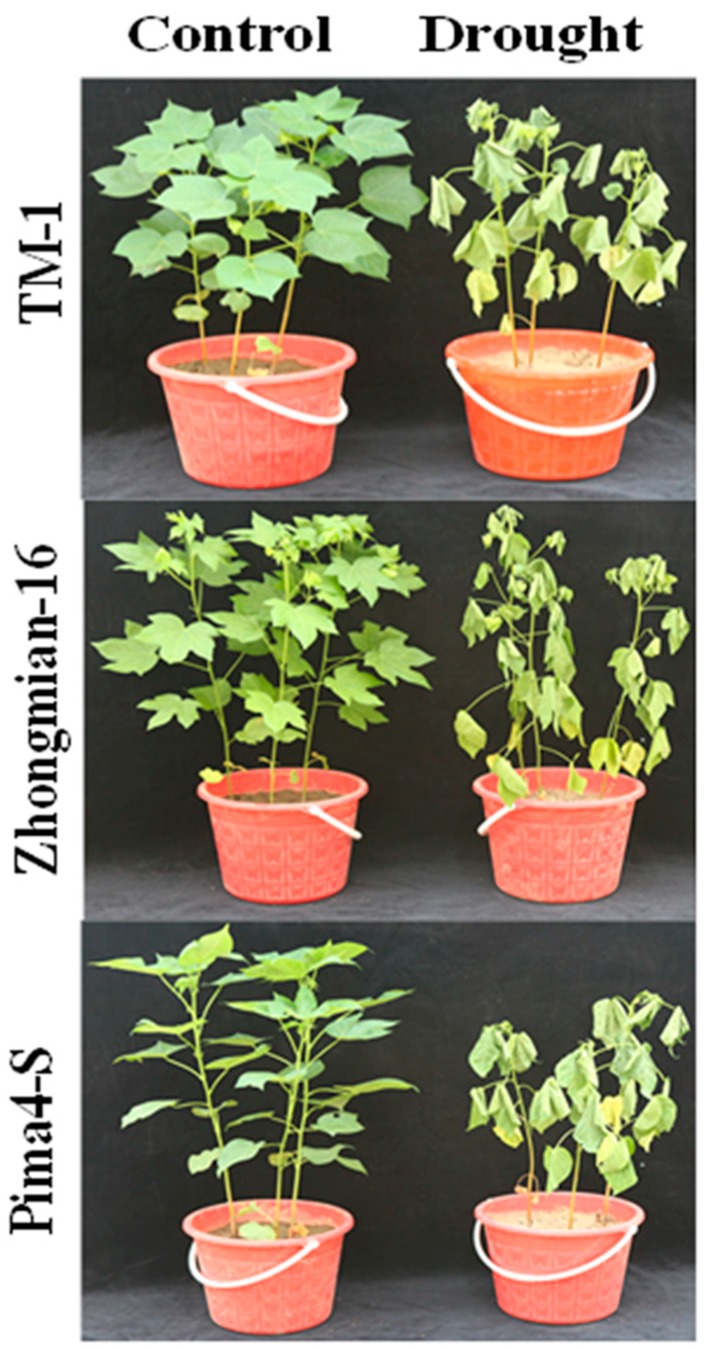
Phenotype of TM-1, Zhongmian-16, and Pima4-S species affected by drought stress.

Drought stress significantly influenced plant height as well as leaf, stem, and fresh and dry root weights in the cotton species analyzed (Table 1). The growth parameters were significantly affected (*p* < 0.05) in all three species (TM-1, Zhongmian-16, and Pima4-S), and the plant exhibited wilting with yellow necrotic patches (Figure 1) due to drought exposure. Earlier studies stated that drought stress seriously inhibits the growth and development of cotton plant [3,49]. Plant height was found to be reduced (10.40%, 15.85%, and 19.31% in TM-1, Zhongmian-16, and Pima4-S, respectively), whereas root length increased (16.88%, 15.17%, and 3.16% in TM-1, Zhongmian-16, and Pima4-S, respectively) under drought treatment. Generally, the root length of plants increases during a water deficit condition because the plants try to acquire underground water to tolerate the stress condition; in line with this, the root length is greater in drought-tolerant species compared to sensitive species [4,5]. Under drought, the leaf, stem, root fresh weights significantly (*p* < 0.05) decreased (14.95%, 16.67%, 27.74% in TM-1, 18.75%, 22.94%, 31.31% in Zhongmian-16, and 25.31%, 55.38%, 53.29% in Pima4-S, respectively), similarly to, the leaf, stem, and root dry weight (7.55%, 7.13%, 18.45% in TM-1, 10.69%, 13.79%, 28.50% in Zhongmian-16, and 16.59%, 24.04%, 41.75% in Pima4-S, respectively) (Figure S1). Under drought stress conditions, the root/shoot ratio significantly (*p* < 0.05) decreased in TM-1, Zhongmian-16, and Pima4-S (13.08%, 17.42% and 27.35%, respectively), concomitantly with a significant (*p* < 0.05) reduction in the relative water content (19.81%, 49.39%, and 58.89%, respectively) (Table 1). Decreased shoot length is a common phenomenon in drought stress, perhaps due to the blockage of vascular tissue vessels and decreased cell elongation, despite a lower reduction in the shoot length being observed in drought-tolerant species than drought-sensitive species [8]. During drought stress, an increased root/shoot ratio is observed in drought-tolerant species under water stress, indicating water acclimatization and increased tolerance [6,7].

Previous studies mentioned that drought is the most critical environmental stress that affects the growth, development, and productivity of plants by changing their morphological growth parameters and biomass accumulation [25,47]. In this study, TM-1 species expressed a higher tolerance than the Pima4-S species, and the Pima4-S species showed a higher sensitivity than the Zhongmian-16 species, as evident in the drought signs visible in Figure 1.

### 2.2. The Effect of Drought on Photosynthetic Parameters, Water Use Efficiency, Chlorophyll Content, and Fluorescence

**Figure 2:**
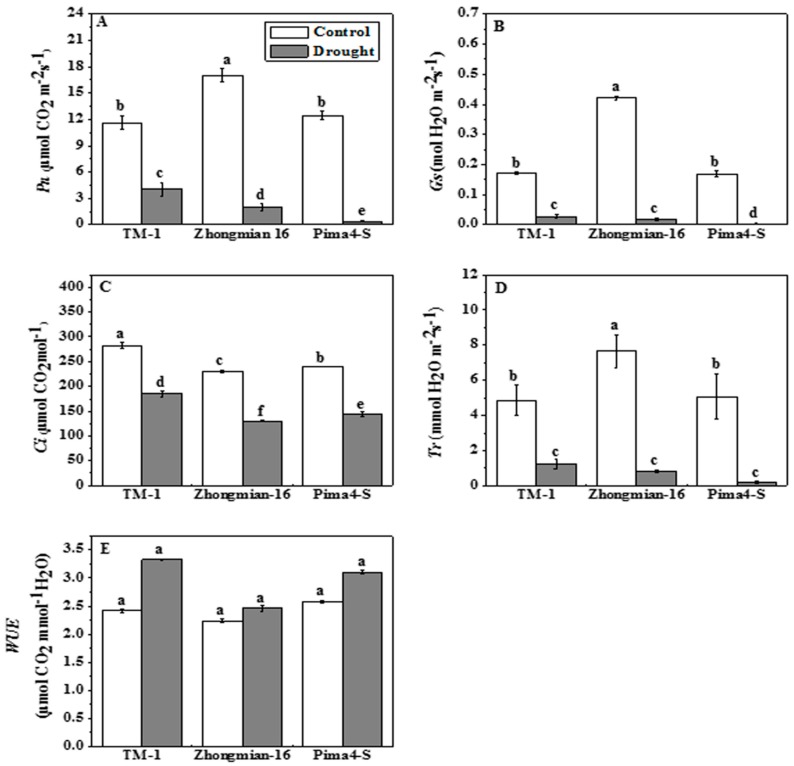
Effect of drought stress on net photosynthetic rate (*P*_n_, **A**); stomatal conductance (*G*_s_, **B**); intercellular CO_2_ concentration (*C*_i_, **C**); transpiration (*T*_r_, **D**) and water use efficiency (WUE, **E**) of the second fully expanded leaves in three cotton species. Means denoted by the same letter are not significantly different at *p* < 0.05. Error bars are ±SD (*n* = 4).

The physiological parameters were affected by the drought stress, but the intensity was different for different species (Figure 2). A significant (*p* < 0.05) percentage reduction in *P*_n_ value of the three cotton species was observed under drought conditions, and the highest *P*_n_ value was recorded for TM-1 (65.20%), followed by Zhongmian-16 (88.27%), and Pima4-S (96.79%) (Figure 2A). The stomatal conductance (*G*_s_) decreased in the three species, and the highest reduction was in Pima4-S (97.87%), whereas the highest *G*_s_ value (83.60%) was recorded in TM-1 (Figure 2B). The *C*_i_ value decreased in drought stress conditions, and a higher reduction was observed in Pima4-S than in Zhongmian-16 and TM-1 (Figure 2C). The drought stress caused a significant (*p* < 0.05) reduction of the transpiration rate (*T*_r_) in all three species (74.94%, 89.45%, and 96.73% in TM-1, Zhongmian-16, and Pima4-S, respectively) (Figure 2D). The water use efficiency (WUE), calculated as the ratio between *P*_n_ and *T*_r_, was higher in the drought-treated plants than in the control plants, but the differences were non-significant (*p* > 0.05) in the three species (TM-1, Zhongmian-16, and Pima4-S, with an increase of 37.74%, 9.69%, and 20.60%, respectively) (Figure 2E). It is reported that photosynthesis is the most important factor that influences crop production under drought stress [50]. Generally, stomatal or non-stomatal factors are involved in the inhibition of photosynthesis [51]. The stomatal closure and decrease in leaf conductance would inhibit the diffusion of CO_2_ to the carboxylation site, as well as decrease photosynthetic uptake. Moreover, the decreased *P*_n_ rate was accompanied by a marked reduction in the *G*_s_, *T*_r_, and *C*_i_ values, indicating that the photosynthesis inhibition caused by these factors (stomatal or non-stomatal) also inhibited chlorophyll synthesis [52]. The total chlorophyll (a + b) content was significantly (*p* < 0.05) decreased in all three species under drought stress conditions (Table 2); Pima4-S was more affected than the other two species (TM-1 and Zhongmian-16). The chlorophyll ratio (a/b) also decreased significantly (*p* < 0.05) under drought by 7.30% in TM-1, 10.45% in Zhongmian-16, and 20.13% in Pima4-S.

**Figure 3:**
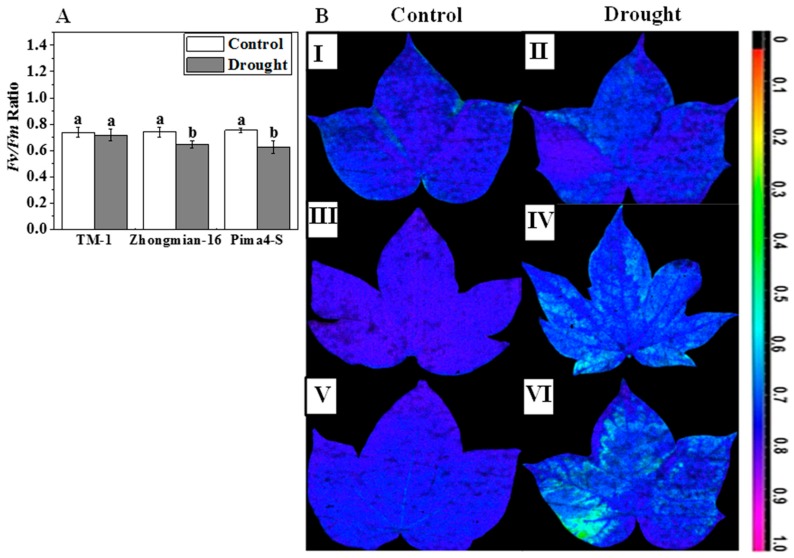
Effect of drought stress on *F*_v_/*F*_m_ Ratio (**A**) of the second fully expanded leaves in three cotton species. Means denoted by the same letter are not significantly different at *p* < 0.05. Error bars are ± SD (*n* = 4); Conversely, false color image (**B**) application was used to understand changes of *F*_v_/*F*_m_ ratio where I, III, V (control) and II, IV, VI (drought treated) leaves of TM-1, Zhongmian-16, and Pima4-S, respectively. In comparison with the control, leaf color shifted from violet blue to light blue along with reducing the *F*_v_/*F*_m_ ratio. The false-color scale depicted the image ranges from 0.2 (yellow) to 1.0 (purple).

The chlorophyll fluorescence parameters (*F*_v_/*F*_m_) was decreased significantly (*p* < 0.05) in Pima4-S (16.92%) and Zhongmian-16 (12.32%), but not in TM-1, in which it was similar to that of the control (2.89% decrease) (Figure 3A). By applying the false color image that explains the changes of the *F*_v_/*F*_m_ ratio by leaf color, the color appeared to be shifted from greenish blue to dark blue in the experimental species. The color intensity gradually increased in Pima4-S, Zhongmian-16, and TM-1 (Figure 3B).

The chlorophyll fluorescence parameter represented by the maximal photochemical efficiency PSII (*F*_v_/*F*_m_) is widely used to determine the photosynthetic activity or efficiency of a plant [13]. A higher reduction of the PSII values indicates a higher sensitivity to drought stress compared to tolerant species that possess a higher protective capacity for PSII as an important tolerance mechanism [53,54]. A remarkable decrease in the *F*_v_/*F*_m_ ratio was observed in all three species during the drought stress, suggesting a possible inhibition of PSII photochemistry by an insufficient energy transfer from the light harvesting chlorophyll complex to the reaction center. The TM-1 species exhibited a more limited reduction of the *F*_v_/*F*_m_ rate compared to the Zhongmian-16 and Pima4-S species. This result might imply that the PSII of Zhongmian-16 and Pima4-S were more sensitive to drought than that of TM-1, which, thus, had a higher protective capacity for PSII.

### 2.3. The Effects of Drought on the Mineral Content, and Na^+^ and K^+^ Concentrations

The mineral content in the leaves was increased under drought stress. Cu increased by 40.93% in TM-1, 38.76% in Zhongmian-16, and 29.87% in Pima4-S. Fe and Mn concentrations increased by 11.84% and 14.90% in TM-1, 4.72% and 3.26% in Zhongmian-16, and 0.76% and 4.39% in Pima4-S. Zn content increased in the leaves of TM-1 (12.50%) and Zhongmian-16 (7.79%), while in Pima4-S it decreased by 1.57% (see Table S1). Under drought, Na^+^ and K^+^ concentrations increased significantly (*p* < 0.05) by 8.76% and 12.33% in TM-1, 4.41% and 6.58% in Zhongmian-16, and 2.07% and 5.51% in Pima4-S (see Table S1). Nutrient ions like Na^+^ and K^+^ play important roles in plant physiology, such as in the regulation of osmosis, the activation of enzymes, and the electrochemical balance through stress signaling and transport mechanisms [55,56]. Recently, it has been proposed that Na^+^ and K^+^ ions have a positive function in response to drought stress, as plants increase the absorption of these ions when subjected to drought stress to overcome the stress-mediated physiological abnormalities [57]. The drought stress was found to induce an increase in Fe [58] and Mn concentrations [25]. High concentration of Zn and Cu were also found in the Ahvaz ecotype of the castor species (*Ricinus communis* L.) in normal conditions [24]. The contents of Fe and Zn in the leaf tissue of *Chlorophytum borivilianum* increased under drought stress [59]. In the current study, drought stress resulted in a larger increase of Cu, Mn, Zn, and Fe concentrations in the leaves of TM-1 than in those of Zhongmian-16 and Pima4-S.

### 2.4. The Accumulation of Proline, Soluble Proteins, and Soluble Sugars during Drought Stress

**Figure 4:**
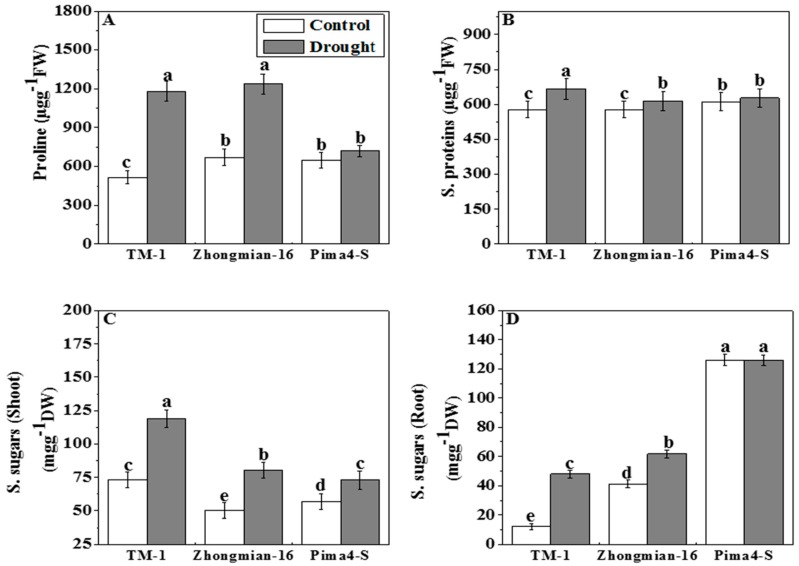
Effects of drought on proline (**A**); soluble proteins (**B**); soluble sugars (Shoot) (**C**) and soluble sugars (Root) (**D**) contents in leaves of three cotton species. Means denoted by the same letter are not significantly different at *p* < 0.05. Error bars are ± SD (*n* = 4).

The Pima4-S genotype exhibited lower proline and soluble sugars content in the shoot and root than the other two cotton species (Figure 4A,C,D). The proline content increased gradually in Pima4-S (10.85%), Zhongmian-16 (84.22%), and TM-1 (129.76%), but the extent of this increase was significantly different (*p* < 0.05) in the three species. Increased soluble proteins were detected in the leaves of the three species (TM-1, Zhongmian-16, and Pima4-S by 15.43%, 6.18%, and 2.67%, respectively) but the increment was non-significant in Pima4-S (Figure 4B). The soluble sugars content in the shoot and root increased significantly (*p* < 0.05) in TM-1 (62.49% and 289.96%) and in Zhongmian-16 (59.56% and 48.28%), while, in Pima4-S, the soluble sugars content increased significantly (*p* < 0.05) in the shoot (28.63%), but almost no change (−0.13%) was detected in the root under drought (Figure 4C,D).

The increasing level of proline content and soluble sugars might be for mitigating drought stress as well as for osmotic adjustment [60]. Sanchez et al. [61] and Alexieva et al. [62] reported that the proline content was increased in peas under water-limited conditions. It has also been reported that the accumulation of soluble sugars in plants is an indicator of higher resistance to drought [63]. The increased molecular protein levels may be for emergency needs and utilized when required for the mitigation of stress and have positive effects on osmotic adjustment [60,64]. In the present study, the proline content and soluble sugars both were increased in TM-1 species compare to the other two species (Zhongmian-16 and Pima4-S) under stress conditions.

### 2.5. The Effect of Drought on Antioxidant Enzyme Activity

**Figure 5:**
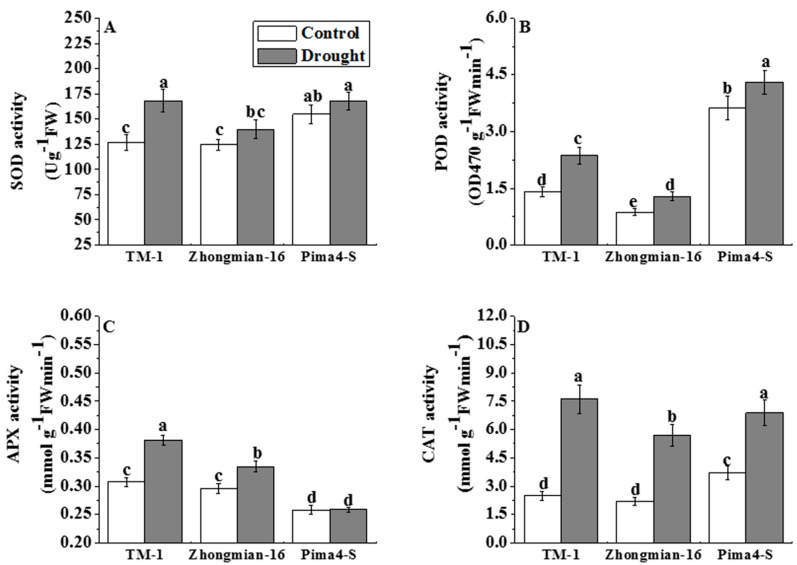
Effects of drought on superoxide dismutase (SOD, **A**); peroxidase (POD, **B**); ascorbate peroxidase (APX, **C**) and catalase (CAT, **D**) activities in leaves of three cotton species. Means denoted by the same letter are not significantly different at *p* < 0.05. Error bars are ± SD (*n* = 4).

The activity of enzymes (SOD, POD, CAT, and APX) involved in ROS scavenging increased significantly (*p* < 0.05) upon drought treatment (Figure 5A–D). SOD activity was higher in TM-1 (32.90%) than Zhongmian-16 (12.58%) and Pima4-S (8.35%) (Figure 5A). POD activity was increased by 68.54% in TM-1, 47.24% in Zhongmian-16, and 18.85% in Pima4-S, and the highest increment was in TM-1 (Figure 5B). The three cotton species showed enhanced CAT activity (TM-1, Zhongmian-16, and Pima4-S by 204.69%, 157.67%, and 86.29%, respectively) under drought conditions, and the highest activity was observed in TM-1 (Figure 5C). A significant (*p* < 0.05) increase in APX activity (Figure 5D) was found in TM-1 (24.09%) and Zhongmian-16 (13.06%), but almost no change was observed in control and drought-treated Pima4-S species (0.16%) under drought stress.

Drought stress is associated with an increase in ROS production in different cell compartments and elevated activities of antioxidant enzymes as an adaptation approach to ameliorate the drought stress-induced oxidative stress [65]. Under stress conditions, ROS homeostasis plays an important role in cellular ionic balance [66]. The ability of plants to overcome oxidative stress relies on the induction of the activities of antioxidant enzymes such as SOD, POD, CAT, and APX [67]. In the present experiment, the enzymes SOD, POD, CAT and APX significantly (*p* < 0.05) increased in TM-1 compared to the other two species (Zhongmian-16, and Pima4-S) under stress conditions.

### 2.6. The Effect of Drought on Lipid Peroxidation, Hydrogen Peroxide (H_2_O_2_), and Superoxide Radical (O_2_^•−^) Content

**Figure 6:**
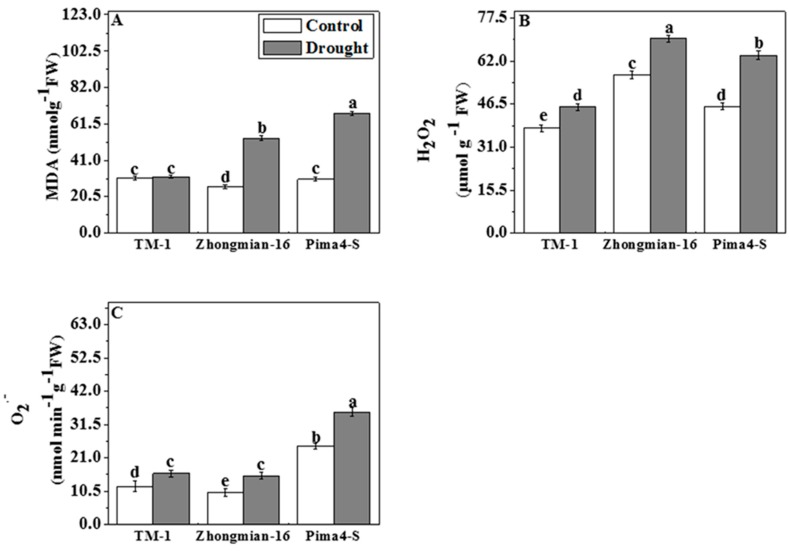
Effects of drought on malondialdehyde (MDA, **A**); hydrogen peroxide (H_2_O_2_, **B**) and superoxide radical (O_2_, **C**) contents in leaves of three cotton species. Means denoted by the same letter are not significantly different at *p* < 0.05. Error bars are ±SD (*n* = 4).

Drought induced a larger production of MDA, H_2_O_2_, and O_2_^•−^ in the leaves of cotton seedlings compared to the control (Figure 6A–C). The MDA content was similar to that of the control in TM-1, but it increased significantly (*p* < 0.05) in Zhongmian-16 (93.07%) and Pima4-S (122.30%) under stress treatment (Figure 6A). H_2_O_2_ and O_2_^•−^ contents increased significantly (*p* < 0.05) in all three species, by 20.04% and 33.25% in TM-1, 22.81% and 51.11% in Zhongmian-16, and 40.30% and 43.42% in Pima4-S, respectively (Figure 6B,C). However, the highest MDA (122.30%) and H_2_O_2_ (40.30%) values were observed in Pima4-S, while the highest O_2_^•−^ concentration (51.11%) was in the Zhongmian-16 species.

Lipid peroxidation has been defined as the oxidative degradation of polyunsaturated lipids [68]. Such an induction of activities of antioxidant enzymes with a lower accumulation of ROS might be evidence of the adaptive potential of plants under drought stress conditions, as mentioned by Petridis et al. [68]. It has been stated that cultivars with a higher drought tolerance have a lower MDA content when exposed to stress [69]. In accordance with increased enzyme activity, the accumulation of H_2_O_2_, O_2_^•−^ and MDA was lower in the TM-1 species compared to Zhongmian-16 and Pima4-S under drought conditions.

### 2.7. Changes in the Expression of Potential Genes during Drought

**Figure 7:**
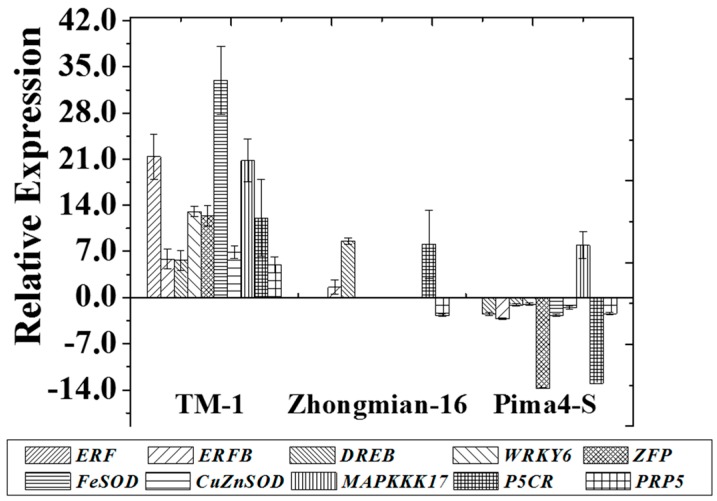
Effect of drought on the mRNA expression level of different drought-responsive genes, transcription factors and other regulatory proteins or enzymes controlling genes. The effects of ethylene responsive factor (*ERF*), ethylene responsive factor binding (*ERFB*), dehydration responsive element binding (*DREB*), *WRKY6*, Zing finger (*ZFP*), Fe superoxide dismutase (*FeSOD*), CuZn superoxide dismutase (*CuZnSOD*), mitogen-activated protein kinase kinase kinase (*MAPKKK17*), pyrroline-5-carboxylate reductase (*P5CR*), proline-rich protein (*PRP5*) genes in the leaves of three cotton species TM-1, Zhongmian-16 and Pima4-S is presented. Total RNAs were extracted from leaves and subjected to reverse transcription followed by real-time PCR. The comparative threshold cycle (*C*_t_) method (2^−^^ΔΔ*C*t^ method) was used to determine the relative gene expression. *EF1α* was used as an internal control. Means denoted by the same letter are not significantly different at *p* < 0.05. Error bars are ± SD (*n* = 3).

To analyze the genetic variation in the examined species under drought stress, the expression level of some selected genes associated with the drought response was studied using RT-PCR. The gene *ERF* (ethylene-responsive transcription factor) was highly expressed in the TM-1 genotype (21.37-fold), while a lower expression was observed in Zhongmian-16 and Pima4-S (0.00 and −2.49-fold, respectively) (Figure 7). The expression of *ERFB* (ethylene-responsive element binding factor) and *DREB* (dehydration responsive element binding protein) genes increased (upregulated) in TM-1, and Zhongmian-16 by 5.88- and 5.65-fold, and 1.55- and 8.61-fold, respectively, but decreased (downregulated) in Pima4-S by −3.20- and −1.10-fold (Figure 7). These two genes might be involved in the drought tolerance of the TM-1 species. Generally, a plant responds to environmental extremes by triggering the expression of genes involved in stress protection [70]. For example, the *ERF* and *ERFB* genes play vital roles in cotton plants under various stress conditions including drought, salinity, hormones, and pathogenic growth responses [35,71,72]. Many researchers also reported that their expression is induced by ethylene, and ABA signaling, as well as abiotic stresses [73]. The *DREB* genes play a significant role as important transcriptional factors that regulate the expression of different stress-responsive genes. In Arabidopsis, the *DREB*s (*CBF3*/*DREB1A*) genes were found to generate a high level of proline and sugars [36], and, in transgenic rice plants, *OsDREB1* increased the soluble sugars level under drought conditions [74]. In TM-1, the *WRKY6* gene expression increased (upregulated) by 13.05-fold, but, in Pima4-S, it decreased (downregulated) by −1.01-fold (Figure 7). Increased expression of the *WRKY6* gene was found in the TM-1 cotton species, which implies that *WRKY6* might be involved in drought tolerance by regulating the expression of osmotic stress-responsive genes (*AtSOS2*, *AtRD29*, and *AtRD29b*) or the ABA signaling pathway [75,76]. *GhWRKY6* gene might be involved in plant resistance against different abiotic stresses. Salt, drought, oxidative and salinity stress have been associated with a rise in SOD activity [77,78]. The expression of *ZFP1* (CCCH-type zinc finger transcription factor) gene increased (upregulated) in TM-1 (12.39-fold), and it was down-regulated in Pima4-S (−13.74-fold). ZFPs play various roles in plant growth and responses to the environment [79,80]. Previously, Luo et al. [81] demonstrated that *GsZFP1* gene is involved in cold and drought tolerance in *Glycine soja*, and Guo et al. [38] showed that the expression of *GhZFP1* increased under drought stress. SODs controlling genes have several isoforms depending on the metal cofactors that bind near the active site, i.e., manganese (*MnSOD*s), copper/zinc (*CuZnSOD*s), iron (*FeSOD*s), and nickel (*NiSOD*s) genes [82]. The expression of the *FeSOD* gene increased (upregulated) 6.92-fold in TM-1, but decreased (downregulated) by −1.50-fold in Pima4-S. The *CuZnSOD* gene expression increased (upregulated) in TM-1 (20.81-fold) and in Pima4-S (7.94-fold). Previously, Negi et al. [39] observed increased activity of the *FeSOD* and *Cu*/*ZnSOD* genes in tobacco under drought stress conditions. The mitogen-activated protein kinase kinase kinase 17 (*MAPKKK17*) gene expression was upregulated by 32.98-fold in TM-1, but was downregulated by −2.66-fold in Pima4-S. Such increased activity of *MAPK* may increase the adaptive potential of plants by regulating various stress signals and hormones like JA, ABA, and ROS homeostasis [40]. In TM-1, *P5CR* (Δ^1^-pyrroline-5-carboxylate reductase) and *PRP5* (proline-rich protein) gene expressions increased (upregulated) by 12.09- and 4.97-fold, while, in Pima4-S, their expression decreased (downregulated) by−13.00- and −2.43-fold, respectively. Many researchers have also reported that the *P5CS*, *P5CR*, and *ProT* genes were upregulated in the reproductive tissues of different plant species under stress condition [83], suggesting a plausible role of proline in flower and reproductive development [84,85]. TM-1 demonstrated a high regulation of all these genes associated with drought tolerance. Our results are also in accordance with those of Kiyosue et al. [86] who observed the up- and downregulation of the enzymes involved in proline biosynthesis and degradation, respectively.

The results of the present study clearly showed that TM-1 accumulated more proline and soluble sugars and demonstrated higher expression of the drought-related gene compared to the other two species. The expression of *ERF* and *ERFB* genes increased significantly in the TM-1 cotton species, as did *ZFP1* gene expression. Furthermore, the protein kinase kinase kinase (*MAPKKK17*) gene also upregulated in the TM-1 cotton species, which indicated a higher drought tolerance level in TM-1 than in the other two species.

### 2.8. Modification in the Ultra-Structure of Chloroplasts in Drought Stress

**Figure 8:**
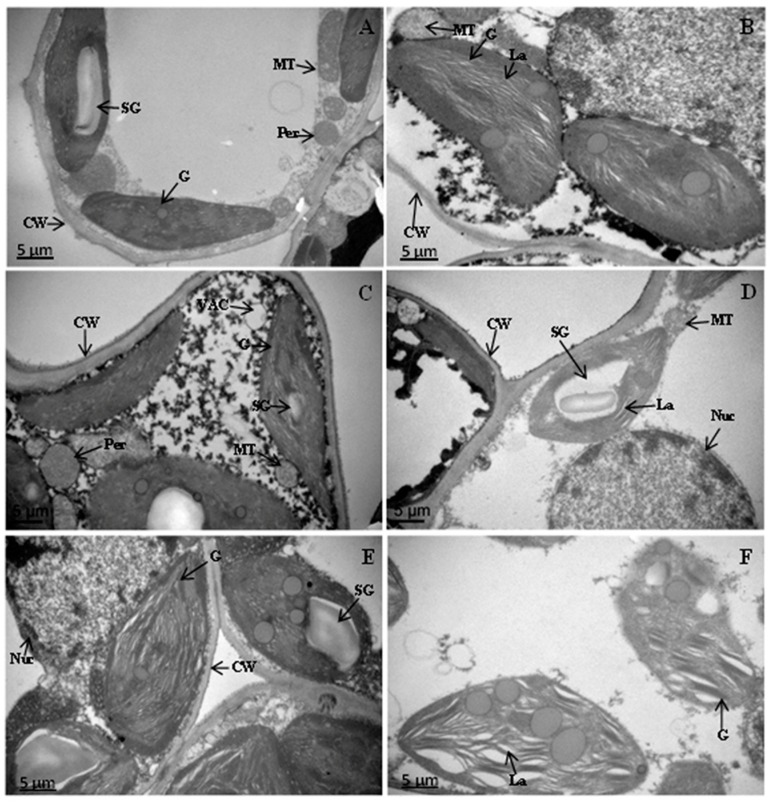
Electron micrographs of leaf mesophyll of cotton species (TM-1, Zhongmian-16 and Pima4-S) under drought stress and control conditions. The TEM micrograph of leaf mesophyll cells of TM-1, Zhongmian-16, and Pima4-S under control shows intact chloroplasts, well-developed grana (G), mitochondria (MC), starch grains (SG) with smooth cell wall (CW), and plasma membrane (PM). (**A**, Control and **B**, drought) TEM micrographs of leaf mesophyll cells of TM-1 show oval shaped chloroplast fewer grana (G), swollen mitochondria (MC) with smooth cell wall (CW); (**C**, control and **D**, drought) TEM micrographs of leaf mesophyll cell of Zhongmian-16 maintained elongated shape of chloroplast with less dense matrix of grana (G) raptured cell wall (CW), starch grain (SG), nucleolus present (Nuc), lamella (La) and mitochondria (MC); (**E**, control and **F**, drought) TEM micrographs of leaf mesophyll cell of Pima4-S show oval shaped chloroplast fewer grana (G), lamella (La). Scale bars represent 5 µm.

The ultra-structural changes in the chloroplasts of leaf mesophylls of the three cotton species under drought stress are shown in Figure 8A–F. The transmission electron micrograph (TEM) of the three cotton species showed well-developed chloroplasts, having closely arranged and packed grana, well-developed mitochondria, and intact organelles in the control condition (Figure 8A,C,E). The TEM micrograph of TM-1 exhibited swollen grana/stroma and abnormal mitochondria with smooth cell walls under drought (Figure 8B). On the other hand, dilated lamellae with large starch grains in the chloroplast and a big nucleus were observed in Zhongmian-16 under drought conditions (Figure 8D). In Pima4-S, reduced grana, dilated lamellae, and loose and broken chloroplast membranes during drought stress were visible (Figure 8F).

The chloroplasts are the common site of the abiotic injury and are visible by ultrastructure observation. The typical changes in the plant ultrastructure in response to drought are damage of the thylakoid membranes, increased number and size of the plastoglobuli, swelling of the thylakoid membranes (stromal and granal), disorganization of the thylakoid membrane system, increase in the intra-thylakoid space, and decrease in the length-to-width ratio and dimensions of the chloroplasts. Drought exposure also changed the shape of many chloroplasts from lenticular to round or oval [87,88]. The reduction of the starch granules can be correlated with glucose starvation upon a decrease in photosynthetic activity [89]. The result of the present studies showed that drought-induced oxidative stress damaged the chloroplasts, leading to the disruption of the thylakoids membrane and chloroplast envelope. However, TM-1 maintained the integrity of the chloroplast and its components like the grana and thylakoids membrane under drought stress conditions. Reduced numbers of starch granules leading to their deficiency in the chloroplasts of Zhongmian-16 and Pima4-S were observed under drought conditions. Nevertheless, the starch accumulated under drought in the TM-1 species, so it is tempting to speculate that starch synthesis plays a vital role in ameliorating the response to drought stress in the cotton plants studied.

## 3. Materials and Methods

### 3.1. Plant Material and Growth Conditions

A pot experiment was conducted at the Zi-jin-gang campus, Zhejiang University, Hangzhou, China, to study the drought effect on the morpho-physiological, biochemical, and molecular characteristics of three cotton species, namely, TM-1 (*Gossypium hirsutum* L.), Zhongmian-16 (*Gossypium arboreum* L.), and Pima4-S (*Gossypium barbadense* L.) in June 2016. The seeds of three cotton species were collected from the Institute of Cotton Research (ICR) Chinese Academy of Agricultural Sciences (CAAS) (Henan, China), and Zhejiang University, Hangzhou, China. The soil was collected from the experimental field of Zhejiang University, Hangzhou, China, and air-dried at 8% moisture content. The air-dried soil was put into plastic pots (7 L, 22 cm in height) and then fertilized with 1 L of a basal nutrient solution, as mentioned by Wu et al. [90]. The solution preparation was as follows (chemical name, µM): Ca(NO_3_)_2_·4H_2_O, 365.2; KNO_3_, 183.0; MgSO_4_·7H_2_O, 547.0; KH_2_PO_4_, 182.2; Fe‒citrate, 19.5; K_2_SO_4_, 91.2; (NH_4_)_2_SO_4_, 365.2; MnCl_2_·4H_2_O, 4.5; ZnSO_4_·7H_2_O, 0.4; CuSO_4_·5H_2_O, 0.2; H_3_BO_3_, 46.9; H_2_MoO_4_, 0.1. The pH of the solution was adjusted to 5.6 ± 0.1 with NaOH or HCl as required. The pots were kept in a net house under natural light. The seeds of all three species were surface-sterilized in 3% H_2_O_2_ for 20 min and rinsed three times with distilled water. The seeds were sown in garden soil on germination trays in the tissue culture lab at a temperature of 30 °C with 50% relative humidity (RH). Uniformly sized, 12-day-old seedlings were transplanted into 7-L plastic pots, three seedlings per pot, and were kept in the net house. Before the drought treatments, the seedlings were irrigated when necessary. The drought started when the plant had 5–6 leaves (30 DAT). The experiment was arranged in a completely randomized design (CRD) with four replications. Plants samples were collected from drought-applied pots when the soil moisture content was 4%. Soil moisture was measured using an HH2Moisture Meter (Delta-T Devices, Cambridge, UK). The plants were gently uprooted and rinsed with running tap water thoroughly. After measuring the plant height and root length, the leaves, stem, and roots were separated, and the fresh weight was measured immediately. The leaves, stem, and roots were then dried at 80 °C for 72 h and weighed.

### 3.2. Analysis of Na^+^, K^+^, and Other Mineral Elements

One hundred mg of dried leaves were powdered and made into ash at 500 °C for 12 h in a muffle furnace. The ash was digested with 5 mL of 30% HNO_3_ and diluted with deionized water [91]. The concentrations of Na^+^, K^+^, and other mineral elements were determined by a flame atomic absorption spectrometer (Shimadzu, AA-6300, Kyoto, Japan).

### 3.3. Determination of the Relative Water Content of the Leaves

The leaf relative water content (RWC) was determined using the following equation proposed by Jones and Turner [92]: RWC (%) = (FW − DW)/(SW − DW) × 100.

Where, FW is the fresh weight, DW is the dry weight, and SW is the saturated weight in water. The dry matter of leaves was determined after drying for 72 h at 80 °C. 

### 3.4. Measurement of Chlorophyll Content, Chlorophyll Fluorescence, Photosynthesis Parameters, and Water Use Efficiency

The chlorophyll content, chlorophyll fluorescence, and photosynthetic parameters were measured on the second uppermost fully expanded leaf with four replicates. The chlorophyll contents were measured according to the method by Arnon [93]. The LI-6400 portable photosynthesis system (LI-COR, Lincoln, NE, USA) was used to measure the net photosynthetic rate (*P*_n_), stomatal conductance (*G*_s_), transpiration rate (*T*_r_), and intracellular CO_2_ concentration (*C*_i_) of the fully expanded second leaf of the plant. The chlorophyll fluorescence parameters were determined using a pulse-modulated chlorophyll fluorometer and the Imaging Win software application (IMAGING-PAM, Walz; Effeltrich, Germany). After 20 min of dark adaptation, the leaves were illuminated under a high saturating light pulse with a frequency of 0.05 Hz for 260 s. The initial fluorescence (*F*_o_) and maximal fluorescence (*F*_m_) were determined using a measuring beam (<0.05 mmol m^−2^s^−1^ PAR and a saturating pulse of 2500 mmol m^−2^s^−1^ PAR, respectively). The variable fluorescence (*F*_v_) was calculated by using the following formula: *F*_v_ = *F*_m_ − *F*_o_. The maximal photochemical efficiency PS II (*F*_v_/*F*_m_) was calculated according to Ahmed et al. [25] by using the Imaging Win software automatically. False color images of *F*_v_/*F*_m_ were recorded, stored, and compared with Imaging Win software. The water use efficiency (WUE) was measured as the ratio between *P*_n_ and *T*_r_.

### 3.5. Determination of Proline, Protein, and Soluble Sugar Contents

Proline was determined according to a modified method by Bates et al. [94]. A fresh leaf sample (0.5 g) was homogenized in 1 mL of 3% (*w*/*v*) aqueous sulfosalicylic acid. Acetic acid (96%) and sulfosalicylic acid (3%) were added in a ratio of 2:1 followed by ninhydrin reagent. The mixture was incubated at 96 °C in a water bath for 1 h and was cooled to room temperature. Before measuring the absorbance, toluene was added, and the toluene aspired from the liquid phase was read at a wavelength of 520 nm. Proline concentration was determined using a calibration curve and expressed as micromole proline g^−1^ FW. Soluble protein concentrations were measured using Bradford assays by following Bradford method [95], briefly, 0.5 g fresh leaf was ground and homogenized in 10 mL of ice–cold potassium phosphate buffer, then centrifuged at 4 °C for 20 min at 12,000× *g*. The supernatant (0.02 mL) was mixed with 2.5 mL reaction solution containing Coomassie Brilliant Blue G-250 (100 mg), which was dissolved in 50 mL of 95% ethanol. To this solution, 100 mL of 85% (*w*/*v*) phosphoric acid was added. The resulting solution was diluted to a final volume of 1 L, and bovine serum albumin was used as a standard. Soluble sugars were estimated by the anthrone reagent method of Yemm and Willis [96]. The anthrone reagent 100 mg, 100 mL H_2_SO_4_, 0.2 mL sample, 0.8 mL distilled water, and 5 mL of mixture solution were heated in a water bath at 95 °C for 10 min, then cooled in ice, and readings were taken by a spectrophotometer (625 nm), as described by Ahmed et al. [25]. For the determination of the total soluble sugars, 0.1 g of a dried sample was dissolved in 10 mL of distilled water and boiled. After cooling, it was transferred to another tube with filter paper, 10 mL of water was added, and the solution was boiled again as before. Then, it was transferred to another tube with filter paper, and finally, water was added to obtain a volume of 25 mL.

### 3.6. Assay of Lipid Peroxidation and Antioxidant Enzyme Activities

The upper second fully expanded leaves were collected for analysis. The leaf sample was cut and weighted, then immediately frozen in liquid nitrogen, and stored frozen at −80 °C for the subsequent analysis of malondialdehyde (MDA) content and antioxidative enzyme activities. The level of lipid peroxidation in the leaf tissue was determined as 2-thiobarbituric acid (TBA) reactive metabolites, chiefly malondialdehyde (MDA), as described by Ahmed et al. [25]. A leaf tissue of 0.2 g was extracted in 1 mL of 0.25% TBA made in 10% trichloroacetic acid (TCA). The extract was heated at 95 °C for 15 min then quickly cooled in an ice bath. After centrifugation at 10,000× *g* for 10 min, the absorbance of the supernatant was measured at 532 nm. Correction of the non-specific turbidity was measured by subtracting the absorbance value taken at 600 nm. The level of lipid peroxidation was expressed using an extinction coefficient of 155 mu cm^−1^. For the determination of antioxidant enzymes activities, samples from the second uppermost fully expanded leaves were homogenized under ice-cold conditions in buffers specific to each antioxidant. Fresh leaves (0.5 g) were ground in liquid nitrogen with a mortar and pestle and then homogenized in 10 mL of ice–cold potassium phosphate buffer (pH 7.0). The mixture was centrifuged at 4 °C for 20 min at 12,000× *g*. The supernatant was used for determining the activity of superoxide dismutase (SOD), peroxidase (POD), catalase (CAT), and ascorbate peroxidase (APX). The total SOD (EC 1.15.1.1) activity was determined according to Giannopolitis and Ries [97] by assessing its ability to inhibit the photochemical reduction of nitroblue tetrazolium chloride (NBT). A 3 mL assay mixture was made using 150 µL of enzyme extract, 50 mM potassium phosphate buffer, 0.1 mM EDTA, 13 mM methionine, 75 µM NBT, and 2 µM riboflavin. The reaction was proceeded by putting the assay tubes under light conditions of 4000 lux for 20 min. Thereafter, the light was turned off, and the tubes were covered with black tissue to terminate the reaction. The non-illuminated solution served as the blank to measure the NBT photoreduction rate (560 nm). One unit of SOD activity was defined as the amount of enzyme required to cause a 50% inhibition of the NBT reduction. POD (EC 1.11.1.7) activity was measured as reported by Zhou and Leul [98] with some modifications. A total of 3 mL of the reaction solution containing 100 µL of enzyme extract mixed with 1.5% (*v*/*v*) guaiacol and 300 mM H_2_O_2_ was prepared in 50 mM potassium phosphate buffer (pH 7.0). Changes in the absorbance related to the oxidation of guaiacol (E = 25.5 mM^−1^ cm^−1^) were measured at 470 nm. The Ascorbate peroxidase (APX, EC 1.11.1.11) activity was measured in 3 cm^3^ of a mixture of 100 mM phosphate buffer (pH 7.8), 0.1 mM Na_2_-EDTA, 0.3 mM ascorbic acid, 0.06 mM H_2_O_2_, and 0.1 cm^3^ of the enzyme extract. The change in absorbance was recorded at 290 nm for 30 s after the addition of H_2_O_2_ [99]. The activity of catalase (CAT, EC 1.11.1.6) was measured according to Aebi [100]. The assay mixture contained 300 mM of H_2_O_2_, 50 mM potassium phosphate buffer (pH 7.8), 2 mM Na_2_-EDTA, and 100 µL of the enzyme extract for a total volume of 3 mL. Absorbance at 240 nm was measured for 1 min (coefficient of absorbance 39.4 mM^−1^ cm^−1^).

### 3.7. Determination of Hydrogen Peroxide, Superoxide Radical

The concentration of hydrogen peroxide (H_2_O_2_) in the leaves was determined according to Willekens et al. [101] with some modifications. The leaves (around 0.3 g) were extracted with 5.0 mL of TCA (0.1%, *w*/*v*) and centrifuged at 12,000× *g* for 15 min. The supernatant (0.5 mL) was carefully taken, and 0.5 mL of phosphate buffer (pH 7.0) along with 1.0 mL of potassium iodide (1 M) was added. The absorbance of the mixture was read at 390 nm. H_2_O_2_ concentration was expressed as l mol g^−1^ FW. Superoxide radical (O_2_^•−^) was determined according to Jiang and Zhang [102] with minor modifications. Fresh leaves (300 mg) were homogenized in 3 mL of 65 mM potassium phosphate buffer (pH 7.8) and centrifuged at 5000× *g* for 10 min at 4 °C. After centrifugation, 1 mL of supernatant was taken and mixed with 0.9 mL of 65 mM potassium phosphate buffer (pH 7.8), and 0.1 mL of 10 mM hydroxylamine hydrochloride and incubated at 25 °C for 20 min. After mixing properly, 1 mL of 17 mM sulfanilamide and 1 mL of 7 mM α-naphthylamine were mixed in a 1 mL solution for further incubation at 25 °C for 20 min. Then, n-butanol (same volume) was added, and the solution was centrifuged at 1500× *g* for 5 min. The absorbance of the supernatant was read at 530 nm. A standard curve was used to calculate the generation rate of O_2_^•−^.

### 3.8. Total RNA Extraction, cDNA Synthesis, and RT-PCR

Total RNA was isolated from the leaves of a plant of each replication within a species (control and drought-treated samples) using the Spectrum™ Plant Total RNA Kit (Easyspin, Merck KGaA, Darmstadt, Germany) according to the manufacturer’s instructions. The cDNA was synthesized from isolated RNA by reverse transcriptase using the cDNA synthesis kit (GoScript™ Reverse Transcription System, Promega, Beijing, China). RT-PCR was performed using the SYBR Green Master Mix (Applied Biosystems™ SYBR™ Green RT-PCR Master Mix, Waltham, MA, USA) with an ABI 7500 Real-Time PCR system (Applied Biosystems^®^ 7500 Real-Time PCR Systems, Waltham, MA, USA). The total reaction volume was 15 μL, containing 1 μL of cDNA, 7.5 μL of 2× SYBR Green Master Mix (Applied Biosystem, Waltham, MA, USA), 1 μL of primer mix (0.5 μM), 0.3 μM of Rox and 5.2 μL of ddH_2_O. All samples were amplified in triplicate assays using the following conditions: 95 °C for 2 min for 1 cycle followed by 40 cycles at 95 °C for 15 s, 55 °C for 30 s, and 72 °C for 30 s and a final extension at 72 °C. The gene-specific primers were designed using PRIMER3 (https://www.ncbi.nlm.nih.gov/tools/primer-blast/). The primer details are listed in Table 3. The cotton *EF1α* (*EF1α*-F: 5′-AGACCACCAAGTACTACTGCAC-3′; *EF1α*-R: 5′-CCACCAATCTTGTACACATCC-3′) gene was used as an endogenous control for all the qRT-PCR analyses. The relative transcription levels were calculated using the 2^−^^ΔΔ*C*t^ method [103].

### 3.9. Analysis of the Ultra-Structural Changes in Chloroplasts

Fully expanded fresh leaves (1 mm^2^) were sectioned by hand and top-middle section was fixed for 6–8 h in 100 mM (pH 7.0) PBS containing 2.5% glutaraldehyde (*v*/*v*), washing three times in the same PBS. The samples were post-fixed in 1% osmium tetroxide (OsO_4_) for 1 h and again washed in PBS for 1 h. Subsequently, the samples were dehydrated in a graded ethanol series (50, 60, 70, 80, 90, 95, and 100%) with 15–20 min intervals followed by acetone (100%) for 20 min, then embedded in Spurr’s resin overnight. Finally, the sections were stained with uranyl acetate and alkaline lead citrate for 15 min. Ultra-thin sections (80 nm) were prepared and mounted on copper grids and viewed under a transmission electron microscope (JEOL JEM-1230 EX, Tokyo, Japan).

### 3.10. Statistical Analysis

The data were analyzed by the SAS 9.3 TS L1M2 program (SAS Institute Inc., Cary, NC, USA). The significance of the differences was determined by the Analysis of Variance (ANOVA) and expressed using Duncan’s multiple range test. The figures containing the mean value and error bars were made using the origin Pro8 software (OriginLab Corporation, Northampton, MA, USA). The figures reporting mean gene expression with error bars were made by using Graph Pad Prism7 software (GraphPad Software, La Jolla, CA, USA).

## 4. Conclusions

The results of this investigation showed that TM-1 had the highest tolerance to drought stress compared to Zhongmian-16 and Pima4-S. Drought stress significantly affected plant growth, chlorophyll content, net photosynthetic rate (*P*_n_), stomatal conductance (*G*_s_), maximum photochemical efficiency PSII (*F*_v_/*F*_m_), and relative water content. The mineral content (Na^+^, K^+^, Fe, Mn, Zn, and Cu) and the accumulation of proline, soluble proteins, soluble sugars (shoot and root), H_2_O_2,_ and O_2_^•−^ significantly increased in the drought-tolerant species (TM-1). The relative expression level of *ERF*, *ERFB*, *DREB*, *WRKY6*, *ZFP1*, *FeSOD*, *CuZnSOD*, *MAPKKK17*, *P5CR*, and *PRP5* genes increased to higher levels in TM-1 than in Zhongmian-16 and Pima4-S under drought stress conditions, conferring higher tolerance to the former species. TM-1 also maintained the integrity of the chloroplasts and their components, like grana and thylakoids membrane under drought stress conditions. Hence, TM-1 can be considered a tolerant genotype that could be employed in the cotton variety development program.

## Figures and Tables

**Table 1:** Effect of drought stress on the growth parameters of three cotton species.

Genotype	Treatment	Plant Height (cm)	Root Length (cm)	Total Fresh Weight (g)	Total Dry Weight (g)	Root Shoot Ratio	Relative Water Content
TM-1	Control	37.25 ± 1.50 ^b^	19.25 ± 1.50 ^d^	21.73 ± 0.21 ^b^	5.38 ± 0.07 ^b^	0.18 ± 0.02 ^a^	83.84 ± 1.61 ^b^
	Drought	33.38 ± 1.11 ^d^	22.50 ± 1.08 ^b^	18.07 ± 0.81 ^c^	4.89 ± 0.02 ^c^	0.15 ± 0.01 ^b^	67.23 ± 2.47 ^d^
Zhongmian-16	Control	41.00 ± 1.15 ^a^	22.25 ± 1.26 ^b,c^	18.69 ± 0.69 ^c^	5.31 ± 0.16 ^b^	0.14 ± 0.01 ^b^	77.81 ± 2.29 ^c^
	Drought	34.50 ± 1.00 ^c,d^	25.63 ± 0.75 ^a^	14.77 ± 0.65 ^e^	4.57 ± 0.14 ^d^	0.12 ± 0.01 ^c^	39.38 ± 2.29 ^e^
Pima4-S	Control	36.25 ± 1.26 ^b,c^	19.75 ± 1.71 ^d^	26.34 ± 0.10 ^a^	6.14 ± 0.12 ^a^	0.18 ± 0.01 ^a^	89.81 ± 4.75 ^a^
	Drought	29.25 ± 1.50 ^e^	20.38 ± 1.11 ^c,d^	16.15 ± 0.75 ^d^	4.72 ± 0.12 ^d^	0.13 ± 0.01 ^b,c^	36.92 ± 0.69 ^e^
LSD_0.05_ between species		1.86	2.10	1.15	0.17	0.02	5.22

Means denoted by the same letter are not significantly different at *p* < 0.05; error bars are ±SD (*n* = 4).

**Table 2:** Effect of drought stress on chlorophyll contents.

Genotype	Treatments	Chlorophyll ^a^	Chlorophyll ^b^	Total Chlorophyll	Chlorophyll Ratio
TM-1	Control	1.85 ± 0.05 ^c^	0.72 ± 0.09 ^b^	2.56 ± 0.14 ^c^	2.61 ± 0.24 ^a^
	Drought	1.53 ± 0.02 ^d^	0.63 ± 0.02 ^c^	2.16 ± 0.02 ^d^	2.42 ± 0.09 ^a,b^
Zhongmian-16	Control	2.29 ± 0.02 ^a^	0.96 ± 0.09 ^a^	3.24 ± 0.09 ^a^	2.39 ± 0.22 ^a,b,c^
	Drought	1.57 ± 0.02 ^d^	0.74 ± 0.02 ^b^	2.31 ± 0.03 ^d^	2.14 ± 0.03 ^c^
Pima4-S	Control	1.97 ± 0.07 ^b^	0.87 ± 0.07 ^a^	2.84 ± 0.14 ^b^	2.27 ± 0.14 ^b,c^
	Drought	1.17 ± 0.01 ^e^	0.65 ± 0.01 ^b,c^	1.82 ± 0.02 ^e^	1.81 ± 0.03 ^d^
LSD_0.05_ between species		0.07	0.11	0.17	0.27

Chlorophyll a, chlorophyll b, total chlorophyll (a + b), and chlorophyll ratio (a/b) of the second fully expanded leaves in three cotton species. Means denoted by the same letter are not significantly different at (*p* < 0.05). Error bars are ±SD (*n* = 4).

**Table 3:** List of primer sequences used for RT-PCR.

Gene Name	Accession No	Forward Primmer (5′ to 3′)	Reverse Primmer (5′ to 3′)
Ethylene responsive transcription factor (*ERF*)	KC222015.1	TCGTGACCCAACGAGGAATG	TGACCGTTGCTTTTCTTGCG
Ethylene responsive element binding factor (*ERFB*)	AY181251.1	CGACAACACCTTCTCGGTGA	TTGTTATCGGCGCTGGTTCT
Dehydration responsive element binding factor (*DREB*)	AF509502.1	GAGGAAGTGGGGAAAGTGGG	CGTATGGAAGCGGCTGAGAT
WRKY6	KF669821.1	CCCCTTTCCCACCATAACCC	AATGGGAATGAAGCAGCGGA
Zinc-finger protein (*ZFP*)	AY887895.1	TCCGTTACATCGTCGTCGTC	AATACGATCACCGCCGTACC
Iron Superoxide dismutase (*FeSOD*)	DQ088821.1	GGCTTGTCAGCTCCTGCTAA	ACGATGATGCACACCCCAAT
CuZn Superoxide dismutase1(*CuZnSOD1*)	AF191342.1	ATGGCTGCATGTCAACTGGA	TGCTCAGTTCATGACCACCC
Mitogen activated protein kinase kinase kinase17 (*MAPKKK17*)	XM_016855464	GTCTCCGGTTTCTTCACGGT	ACCTCCGGTGCCATGTAAAG
pyrroline-5-carboxylate reductase	XM_016889238	GCAACAGAACAGGATGGGGA	CACTAAGGCCAGTGACAGCA
Proline rich protein (*PRP5*)	EF095706.1	TACGACGAGAAGGCCAACAC	TTGGGCTTTGGAGGTGGTTT

## Supplementary Materials

The following are available online at http://www.mdpi.com/1422-0067/19/9/2636/s1, Figure S1: Effect of drought stress on fresh weight and dry weight of leaves, fresh weight and dry weight of stem, fresh weight and dry weight of root, in three-cotton species, Table S1: Na+, K+ and other mineral elements of three cotton species exposure to drought.
