# Supplementary material for: Molecular and Physio-Biochemical Characterization of Cotton Species for Assessing Drought Stress Tolerance
Source: Int J Mol Sci. 2018 Sep 6;19(9):2636. doi: 10.3390/ijms19092636 (PMC6163957; doi:10.3390/ijms19092636)
Supplement: Supplementary file 1 [file ijms-19-02636-s001.pdf]

International Journal of
Molecular Sciences

MDPI

Supplementary information

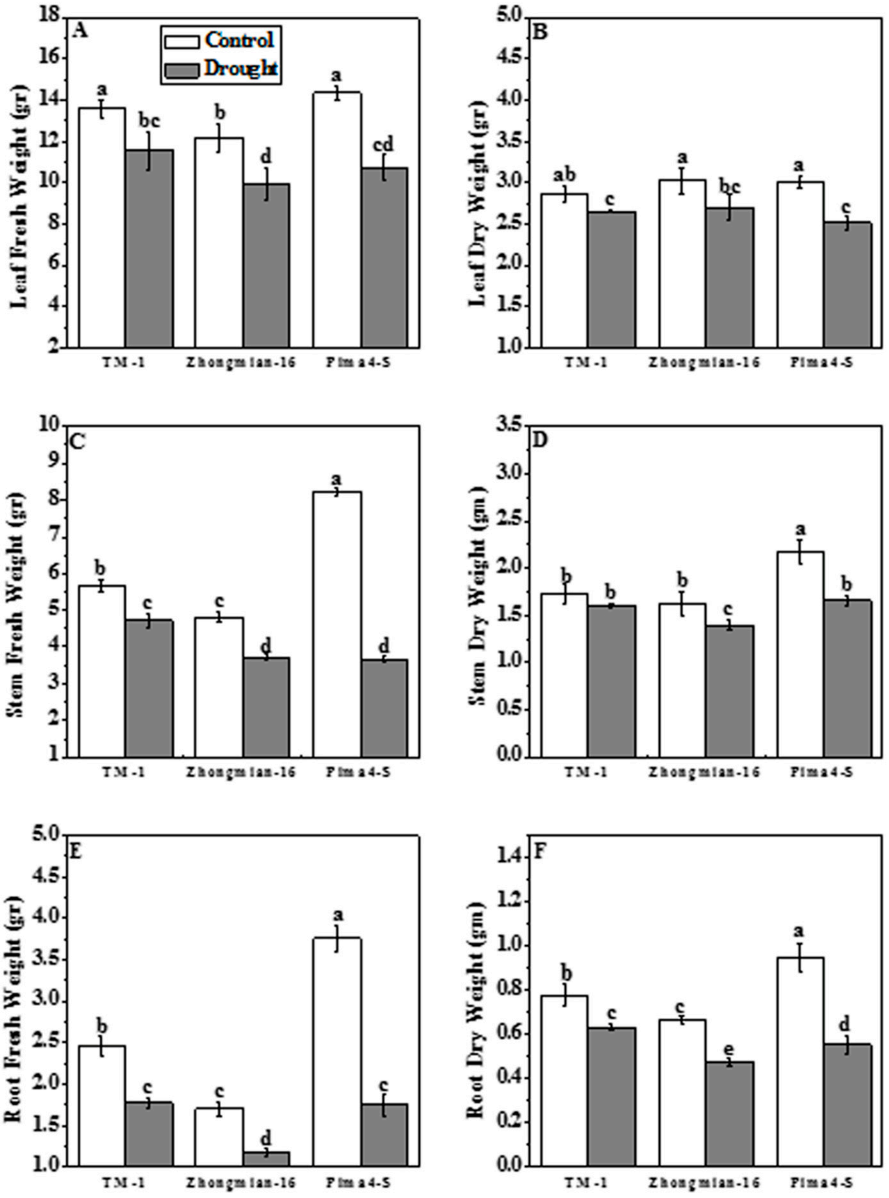

Figure S1: Effect of drought stress on fresh weight and dry weight of leaves, fresh weight and dry weight of stem, fresh weight and dry weight of root, in three-cotton species. Means denoted by the same letter are not significantly different at $p < 0.05$. Error bars are ± SD ($n = 4$).

Table S1: Na^{+}, K^{+} and other mineral elements of three cotton species exposure to drought.

Genotype	Treatment	Mineral content (mg g^{-1} DW)					
		**Na**	**K**	**Fe**	**Mn**	**Zn**	**Cu**
**TM-1**	**Control**	1.16 ± 0.04[b]	20.03 ± 0.51[e]	0.24 ±0.02[b]	0.05 ±0.00[b]	0.03± 0.00[bc]	0.01 ± 0.00[b]
	**Drought**	1.26 ± 0.02[a]	22.50 ± 0.08[c]	0.27 ±0.03[a]	0.05 ± 0.00[a]	0.04 ± 0.00[a]	0.02 ± 0.00[a]
		(+8.76%)	(+12.33%)	(+11.84%)	(+14.90%)	(+12.50%)	(+40.39%)
**Zhongmian-16**	**Control**	0.79 ± 0.01[c]	24.24 ± 0.39[b]	0.20 ±0.02[c]	0.04 ± 0.00[b]	0.03 ± 0.00[cd]	0.01 ± 0.00[b]
	**Drought**	0.82 ± 0.06[c]	25.83 ± 0.07[a]	0.21 ±0.01[bc]	0.04 ± 0.00[b]	0.03 ± 0.00[b]	0.02 ± 0.00[a]
		(+4.41%)	(+6.58%)	(+4.72%)	(+3.26%)	(+7.79%)	(+38.76%)
**Pima4-S**	**Control**	0.36 ± 0.03[d]	21.31 ± 0.33[d]	0.20 ±0.01[c]	0.04 ± 0.00[b]	0.03 ± 0.00[cd]	0.01 ± 0.00[b]
	**Drought**	0.37 ± 0.01[d]	22.49 ± 0.27[c]	0.20 ±0.01[c]	0.04 ± 0.00[b]	0.03 ± 0.00[d]	0.02 ± 0.00[a]
		(+2.07%)	(+5.51%)	(+0.76%)	(+4.39%)	(-1.57%)	(+29.87%)
**$LSD_{0.05}$ between species**		0.06	0.62	0.03	0.01	0.00	0.00

Results are presented as the mean of 4 individual measurements. Means denoted by the same letter are not significantly different at $p < 0.05$. Error bars are ± SD ($n = 4$). Values in parenthesis are expressed as a decreased (-)/increased (+) percentage of the control.
